# Ecosystem metabolism drives pH variability and modulates long-term ocean acidification in the Northeast Pacific coastal ocean

**DOI:** 10.1038/s41598-018-37764-4

**Published:** 2019-01-30

**Authors:** Alexander T. Lowe, Julia Bos, Jennifer Ruesink

**Affiliations:** 1 Present Address: Tennenbaum Marine Observatories Network, Smithsonian Institution, 647 Contees Wharf Road, Edgewater, MD 21307 USA; 2 Department of Biology, University of Washington, 24 Kincaid Hall, Seattle, WA 98195 USA; 3 Washington Department of Ecology, 300 Desmond Dr. SE, Lacey, WA 98503 USA

## Abstract

Ocean acidification poses serious threats to coastal ecosystem services, yet few empirical studies have investigated how local ecological processes may modulate global changes of pH from rising atmospheric CO_2_. We quantified patterns of pH variability as a function of atmospheric CO_2_ and local physical and biological processes at 83 sites over 25 years in the Salish Sea and two NE Pacific estuaries. Mean seawater pH decreased significantly at −0.009 ± 0.0005 pH yr^−1^ (0.22 pH over 25 years), with spatially variable rates ranging up to 10 times greater than atmospheric CO_2_-driven ocean acidification. Dissolved oxygen saturation (%DO) decreased by −0.24 ± 0.036% yr^−1^, with site-specific trends similar to pH. Mean pH shifted from <7.6 in winter to >8.0 in summer concomitant to the seasonal shift from heterotrophy (%DO < 100) to autotrophy (%DO > 100) and dramatic shifts in aragonite saturation state critical to shell-forming organisms (probability of undersaturation was >80% in winter, but <20% in summer). %DO overwhelmed the influence of atmospheric CO_2_, temperature and salinity on pH across scales. Collectively, these observations provide evidence that local ecosystem processes modulate ocean acidification, and support the adoption of an ecosystem perspective to ocean acidification and multiple stressors in productive aquatic habitats.

## Introduction

Nearshore aquatic ecosystems are changing rapidly as a function of altered riverine inputs^1^, eutrophication^2^ and loss of foundation species due to human influence^3,4^. Collectively these physical and ecological processes are changing carbon cycling, and interact with atmospheric CO_2_ via warming and acidification to influence ecosystem function^5,6^. While elevated aqueous CO_2_, and the concomitant decrease of pH, has been shown to have negative effects across marine phyla^7^, the influence of long-term anthropogenic CO_2_ emissions and the response of biological communities may vary by habitat in relation to local biotic and abiotic conditions^8–10^. Microbial respiration has long been recognized as a driver of CO_2_ exchange with the atmosphere along the aquatic continuum^11,12^, particularly in net heterotrophic habitats such as shallow tidal estuaries and fjords that are typically sources of CO_2_ to the atmosphere^6,12^. Conversely, continental shelf and seasonally productive ecosystems can be CO_2_ sinks^13^, suggesting a mechanistic link between local water chemistry and ecological factors such as interactions with organisms that modify the local environment, e.g. ecosystem engineers^14^, and food web dynamics^15^. While studies have shown that biological feedbacks have strong effects on carbonate chemistry on short time and small spatial scales^16,17^, empirical studies investigating the potential for local ecological modulation of long-term global ocean acidification are lacking.

Currently, organisms are regularly exposed to ‘future’ levels of lower pH in nearshore ecosystems^18,19^. The implications of these periods of low pH may depend on the timing of exposure^20^, and go beyond the isolated physiological effects of pH when accompanied by multiple stressors like low oxygen or thermal stress^21^. But what controls these pH changes? Seawater pH has been demonstrated to respond to local community composition^9,16,22^ and changes to water column productivity or suspended particulate matter composition^23,24^ through the balance of primary productivity and respiration, referred to as net ecosystem metabolism. Few studies have paired quantification of ecosystem processes with pH, yet dissolved oxygen is commonly measured as an indicator of net ecosystem metabolism^25^. Long-term trends of pH that cannot be explained by increasing atmospheric CO_2_ have been observed in coastal systems^26,27^ including long-term increases in mean pH^8,28^. Empirical studies that compare trends in metabolic state and pH may fill this knowledge gap. The interaction of biological effects with water mass transport and physical processes could explain local variability of seawater pH on decadal^10^, annual^29^ and monthly to hourly scales^18,30^. Addressing ocean acidification from an integrated ecological framework may help define the temporal and spatial structure of exposure to pH stress and the relationship to other energetic and ecological factors at scales relevant to populations and communities^31^.

**Figure 1:**
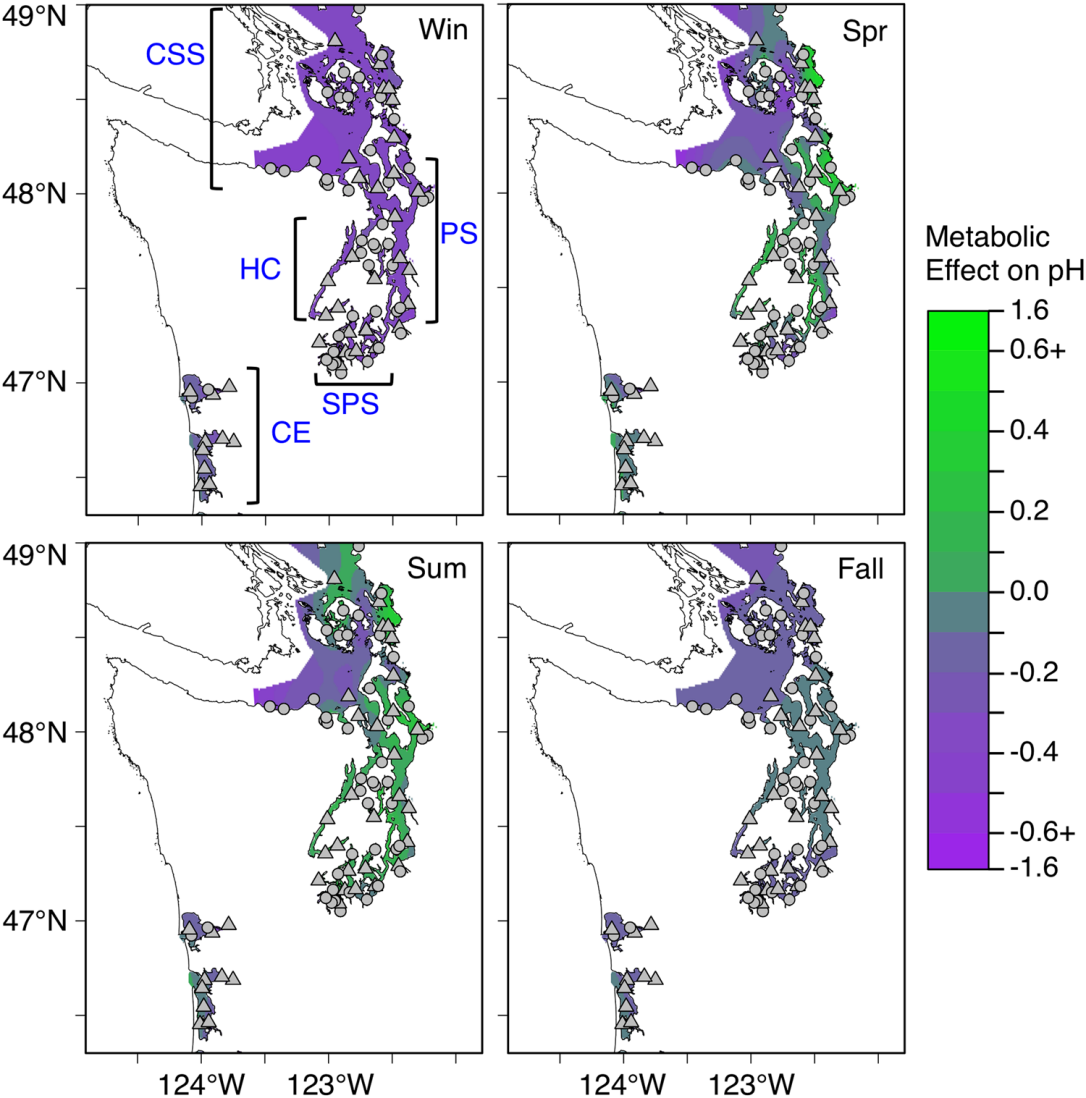
Seasonal mean metabolic effect on pH (observed pH minus predicted ‘atmospheric equilibrium’ pH) in Washington state waters, USA. Sampling location identified by dots (rotating) and triangles (core). Subregions defined in the analysis are coastal estuaries (CE), Hood Canal (HC), South Puget Sound (SPS), Puget Sound (PS) and Central Salish Sea (CSS).

Previous studies have used long-term monitoring datasets to show that patterns of pH in shallow estuaries are closely linked to dissolved oxygen^10^ and to hypothesize that long-term pH changes in coastal shelf ecosystems are related to ecosystem productivity^28^. In the current study, we quantified spatial and temporal seawater pH dynamics within a Northeast Pacific coastal region that includes upwelling-influenced shallow coastal estuaries, deep seasonally stratified fjord-like embayments, and tideflats. The diversity of habitats sampled in this study is representative of many seasonally productive coastal habitats around the world. We used monthly observations of water properties collected by U.S. state and federal agencies over 25 years to test the relative importance of hypothesized drivers of pH variability at 83 sites across ~7500 km^2^ of coastal ecosystems in Washington state (Fig. 1). This region supports valuable wild and aquaculture shellfish industries that make up 45% of shellfish aquaculture in the US^32^ and may be vulnerable to long-term carbonate chemistry changes^33^. We compared long-term trends of pH (NBS scale) to atmospheric CO_2_ concentrations, then tested the hypotheses that global ocean acidification is modulated by local changes in net ecosystem metabolism as indicated by dissolved oxygen saturation. We then compared pH to physical and biological variables to test the relative importance of these variables at multiple timescales.

## Results

### Spatial and temporal variation of pH and %DO

#### Multi-decadal pH and %DO trends

**Figure 2:**
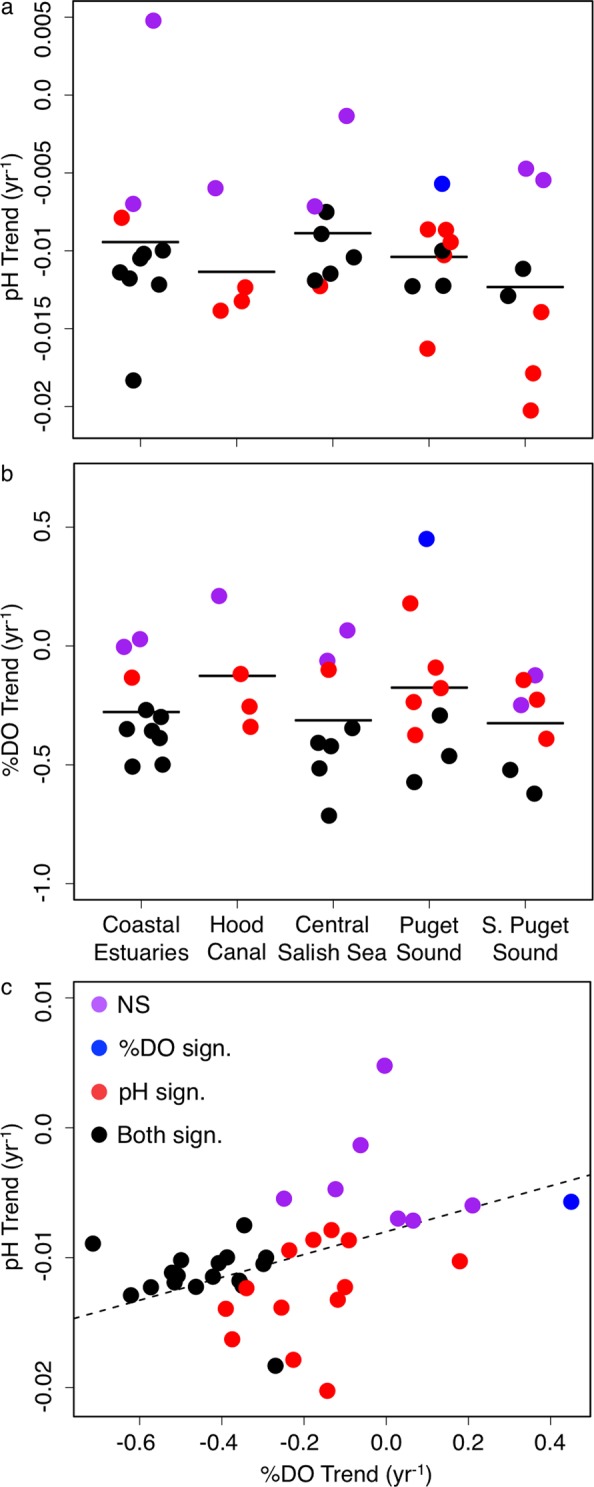
Calculated pH (**a**) and %DO (**b**) change per year for each subregion (horizontal bars) and individual core sites (points) from 1991–2015. Subregions for a and b shown below Fig. 2b. Site-specific trend of pH vs. trend of %DO (**c**). Dashed line is the slope of regression fit to all pH vs. %DO observations. Color of point indicates significance of trend: NS = neither pH or %DO trend over time were significantly different than zero at that site, DO Sign. = only %DO trend significant, pH Sign. = only pH trend significant, Both Sign. = both trends significant. Number of observations varies by site.

Ecosystem-wide mean seawater pH decreased significantly during the period of sampling at a rate of −0.009 ± 0.0005 pH yr^−1^ (LME: *X*^2^ = 331.5, *df* = 1, p < 0.001). Estimates of mean pH trend showed little variation (−0.009 ± 0.0001 pH) associated with the method of subsampling data to unify datasets. Annual lower quartile pH declined significantly at −0.008 ± 0.003 pH yr^−1^ over the sampling period (linear regression: *t*_1,23_ = −2.78, *r*^2^ = 0.22, *p* = 0.011), whereas annual upper quartile pH did not change over the period (linear regression: *t*_1,23_ = −0.75, *r*^2^ = 0.023, *p* = 0.459). The mean pH decreased significantly at 30 of 38 core sites at rates ranging from −0.008 to −0.020 pH yr^−1^ (Fig. 2a). The mean rate of pH change varied by subregion, and was greatest in South Puget Sound. %DO decreased in the region by −0.24 ± 0.036% yr^−1^ (LME: *X*^2^ = 42.2, *df* = 1, p < 0.001). Significant negative trends were observed at 17 of 38 core sites and ranged from −0.27 to −0.71% yr^−1^, with one significant positive trend of +0.45% yr^−1^ (Fig. 2b). Site-specific trends of pH and %DO were similar to the expected relationship derived from the regression of pH to %DO (Fig. 2c; slope = 0.008), but deviations at multiple sites lead to a non-significant relationship between the trends.

#### Seasonal and spatial variability of pH and %DO

**Figure 3:**
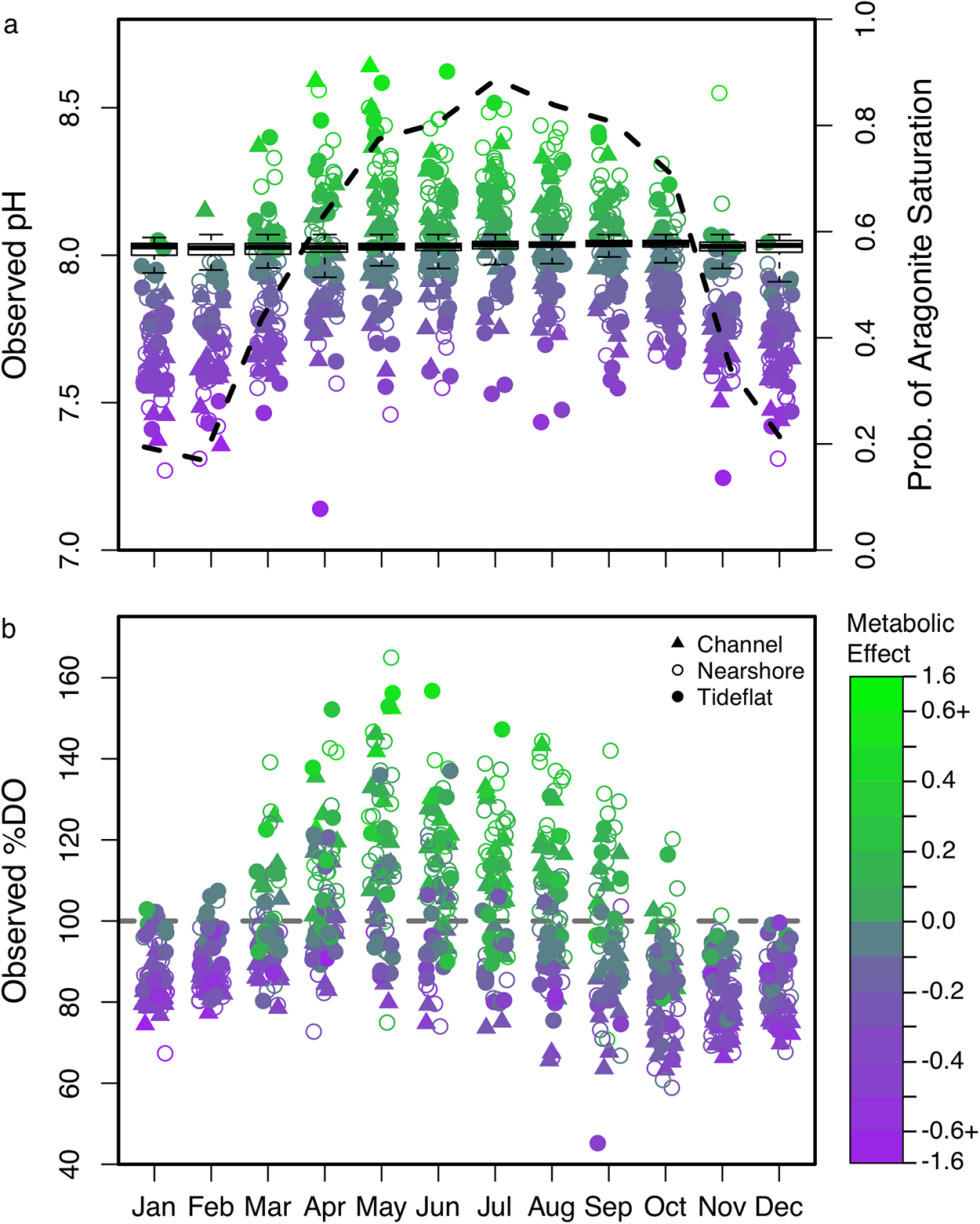
Seasonal variation of observed pH and %DO. (**a**) Mean monthly site-specific pH (points), distribution of predicted ‘atmospheric equilibrium’ pH across all sites for each month (boxplots), and the probability of observing aragonite supersaturation within the entire sampling region in a given month (dashed line corresponding to right axis). (**b**) Mean monthly site-specific dissolved oxygen saturation. Color of points in a and b corresponds to the magnitude of the metabolic effect on pH (observed pH minus ‘atmospheric equilibrium’ pH) as indicated in the legend (same as Fig. 1). Shape of points indicates habitat of the site: triangle = channel, open circle = nearshore, filled circle = tideflat.

Overall, mean daytime pH was 7.92 ± 0.30 in Washington State surface waters during the period from 1991 to 2015 (Table 1). Average %DO was 97.8 ± 21.5%. The probability of observing aragonite supersaturation (Ω > 1) shifted from <20% in winter to 88% in July (Fig. 3a), concomitant to a system-wide shift from net heterotrophy (%DO < 100%) to autotrophy (%DO > 100%). The seasonal oscillation of median pH was robust enough to be observed even though mean pH decreased by ~0.22 units over the period of sampling. Metabolic contribution to pH varied considerably among sites and seasons (Fig. 1), leading to a range of pH as much as 1.3 units above and below predicted atmospheric equilibrium. These changes were much greater than could be explained by physical factors alone (black boxplots show distribution of ‘atmospheric equilibrium’ pH; Fig. 3a). In total, only 6.6% of observations over 25 years were within one standard deviation of the mean ‘atmospheric equilibrium’ seawater pH.

**Table 1:** Summary of mean and standard deviation of pH observations by season and habitat.

	Obs.	Channel	Nearshore	Tideflat	System-wide
		2368	2042	1585	5995
Win	1066	7.61 ± 0.21	7.69 ± 0.23	7.80 ± 0.23	7.69 ± 0.23
Spr	1738	7.91 ± 0.29	8.00 ± 0.29	7.99 ± 0.31	7.96 ± 0.30
Sum	1766	8.03 ± 0.24	8.11 ± 0.25	7.94 ± 0.32	8.03 ± 0.27
Fall	1425	7.85 ± 0.22	7.93 ± 0.24	7.88 ± 0.27	7.89 ± 0.24
Annual	5995	7.88 ± 0.28	7.96 ± 0.30	7.91 ± 0.30	7.92 ± 0.29

Number of observations in each category shown in table.

Significant spatial and temporal variation of mean pH and %DO was observed at multiple scales (Table 2); pH and %DO varied significantly among sites and months (Fig. 3), as well as at the subregional and seasonal scale (Table 2). Habitat was also a significant predictor of pH and %DO (Table 2; Fig. 3). The pH at channel and nearshore sites exhibited high variation among seasons, on average ranging 0.43 pH from summer to winter in channel and 0.42 pH in nearshore sites (Table 1). Tideflats exhibited high pH variation within seasons, particularly in summer and fall when variation was ~30% and 17% higher, respectively, than channel and nearshore habitats (Bartlett’s test: Summer, *K*^2^ = 56.4, *p* < 0.001; Fall, *K*^2^ = 18.3, *p* < 0.001; Table 1). Seasonal variation over the year was largely due to changes in the maximum observed pH, which varied >1.2 pH from winter to summer, rather than a change in the minimum observed pH which varied ~0.3 pH over the year. %DO exhibited a similar pattern (Fig. 3b)

**Table 2:** Results of linear models comparing pH and %DO across spatial and temporal scales.

Variable	*pH*			*%DO*		
	*Df*	*F*	*p*	*Df*	*F*	*p*
Site	82	25.30	<0.001	82	30.46	<0.001
Month	11	216.10	<0.001	11	311.87	<0.001
Site*Month	794	2.09	<0.001	794	2.95	<0.001
Residuals	5107			5107		
Subregion	4	44.05	<0.001	4	65.33	<0.001
Season	3	419.91	<0.001	3	560.59	<0.001
Subregion*Season	12	27.62	<0.001	12	40.04	<0.001
Residuals	5975			5975		
Habitat	2	47.67	<0.001	2	52.28	<0.001
Season	3	409.76	<0.001	3	516.96	<0.001
Habitat*Season	6	27.93	<0.001	6	28.44	<0.001
Residuals	5983			5983		

#### Time of day

Surface pH increased 0.026 ± 0.001 pH hr^−1^ (LME: *X*^2^ = 250.41, *df* = 1, p < 0.001) and %DO increased 1.54 ± 0.010% hr^−1^ (LME: *X*^2^ = 132.4, *df* = 1, p < 0.001) during the daytime sampling period when controlling for interannual and subregional differences.

### Drivers of pH variability

**Figure 4:**
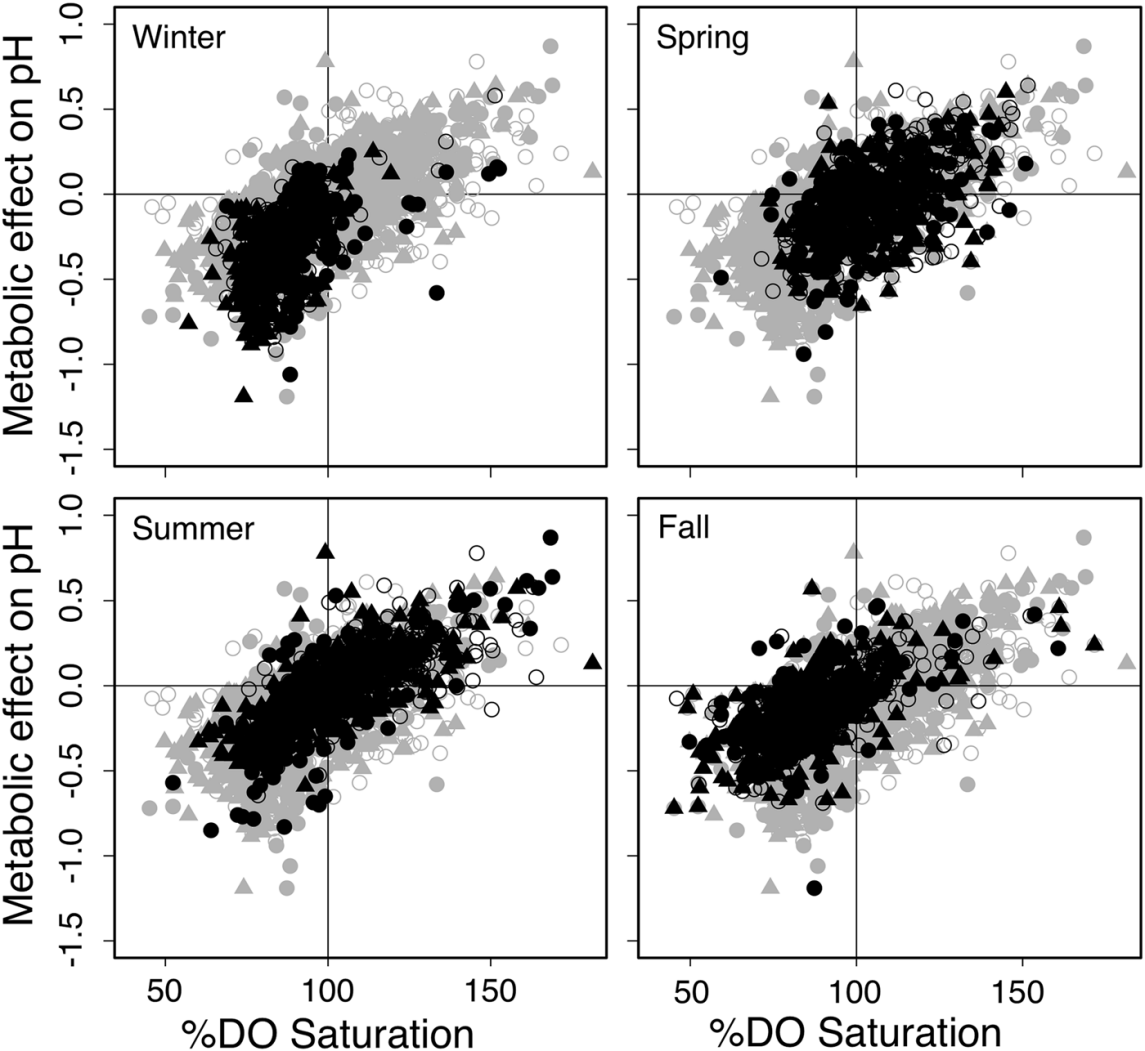
Relationship of mean seasonal metabolic effect on pH vs. %DO saturation. Crossbars indicate ‘atmospheric equilibrium’ pH and 100% DO saturation. Black points represent the seasonal site-specific mean pH across all years of sampling. Light gray points show all values. Shape of points indicate habitat: triangle = channel, open circle = nearshore, filled circle = tideflat.

pH was significantly positively correlated to dissolved oxygen, temperature and salinity, whereas pH was significantly negatively correlated to atmospheric CO_2_ (Table 3). The scaled estimate of %DO influence on pH was nearly three times larger than the effect of temperature (0.595 vs. 0.184) and an order of magnitude greater than the effect of salinity (0.030). The effect of atmospheric CO_2_ was of similar magnitude but opposite sign as temperature (−0.201). %DO alone explained 50.9% of the variation in pH, whereas the full model including temperature, salinity and atmospheric CO_2_ explained 54.8%. Chlorophyll was positively correlated to pH in the subset of data with chlorophyll measurements (Supplementary Table 2). The relationship between pH and DO varied slightly by season (Fig. 4); the slope of the regression of pH anomaly to %DO was 0.011 in winter (r^2^ = 0.27, p < 0.001), 0.008 during spring (r^2^ = 0.28, p < 0.001), 0.009 in summer (r^2^ = 0.51, p < 0.001) and 0.008 in fall (r^2^ = 0.39, p < 0.001). Both DO and pH were predominately lower than atmospheric equilibrium in fall and winter, with a large shift towards positive pH anomalies and DO supersaturation occurring in spring and summer (Fig. 4).

**Table 3:** Results of linear mixed effects model comparing pH variation to metabolic (%DO), physical (temperature and salinity) and climatic (atmospheric CO_2_) drivers.

	Estimate	SE	*X* ^2^	*p*
DO % Saturation	0.595	0.010	2672.30	<0.001
Atmosphere CO_2_	−0.201	0.009	507.86	<0.001
Temperature	0.184	0.014	157.62	<0.001
Salinity	0.030	0.010	7.94	0.005

(see methods for details, briefly: LME model with 5994 observations scaled to (x-µ)/SD for each variable. Fixed effects shown, random effects were Subregion and Season.

## Discussion

Multi-decadal changes of surface water pH have been observed in marine ecosystems^34,35^ yet few datasets have quantified the parameters necessary to test hypotheses about the potential for biological processes to modulate pH changes over time (but see^10^). We quantified the effects of atmospheric CO_2_, physical and biological factors on seawater pH using a unique dataset spanning 25 years of sampling at 83 sites representing shallow estuarine, nearshore, and deep, stratified coastal habitats. Surface seawater pH decreased significantly over the 25-year study period in this NE Pacific coastal ecosystem. While pH was negatively correlated to atmospheric CO_2_, the rate of pH decrease was on average ~5 times greater than that predicted from atmospheric CO_2_ changes alone. Concomitant changes to dissolved oxygen support the hypothesis that local biological processes modulated global ocean acidification, in this case accelerating long-term pH decline. Multiple lines of evidence support the hypothesis that changes to local ecosystem metabolism and a system-wide shift towards greater net heterotrophy, more than physical oceanographic conditions or atmospheric CO_2_, caused the rapid decline of pH. First, the ecosystem-wide trend of −0.009 pH yr^−1^ corresponded to deoxygenation at a rate of −0.24% yr^−1^. Second, the rate of pH and %DO were non-uniform among sites within the same ecosystem, with significant pH decreases ranging from 4–10 times that of open ocean rates of acidification. Third, this system was on average a source of CO_2_ to the atmosphere and pH was within one standard deviation of atmospheric equilibrium only ~6% of the time. Fourth, %DO explained a majority of variation in pH across scales, including coherent seasonal variation across the region and observable increases associated with the time of day samples were collected. Finally, the significant long-term decline of pH was driven by changes to the lower quartile of pH from an intensification of heterotrophic conditions with no corresponding change to the upper quartile range, contrary to model predictions driven by atmospheric changes^17^. Collectively, these observations implicate local ecosystem metabolism as the main driver of variation of pH in NE Pacific coastal habitats. The diversity of habitats represented in this analysis indicates these findings are generalizable to many coastal ecosystems.

This empirical analysis of long-term pH and oxygen changes revealed a primary influence of net ecosystem metabolism on pH dynamics. Biological community composition has been observed to influence carbonate chemistry variation in tide pools and coral reef flats^9,16^; we provide evidence that strong biological influence can extend to an ecosystem-scale. The extremes of pH (6.5 < pH < 9.3) were far outside the range driven by physical changes alone and were correlated to extremes of %DO. The widespread prevalence of respiration-driven undersaturation of surface waters in fall and winter means organisms are regularly exposed to stressful carbonate chemistry conditions as a function of the seasonal cycle of biomass production and decomposition. Yet as %DO increased seasonally, aragonite saturation state shifted from levels stressful to local organisms^7,36,37^ to benign conditions. The shift towards benign conditions corresponded to the timing of sensitive early life history stages in many organisms^38^, suggesting these seasonal fluctuations may have considerable consequences for populations of aquatic organisms. The system-wide, biologically-driven shift towards higher pH in summer is likely an important feature of seasonally productive nearshore ecosystems.

The observed habitat-specific pH dynamics lend further support for the importance of considering ecological processes in ocean acidification studies, and the need for studies parsing variability among benthic and pelagic factors. High macrophyte biomass, often cited as a potential acidification refuge^39^, may have contributed to elevated pH in tideflat habitats, particularly in eelgrass-dominated areas of PB-NERR (Central Salish Sea) and Willapa Bay (Coastal Estuaries). Macrophyte production can exert large positive effects on pH in shallow coastal habitats^10,39,40^, but could not explain elevated pH observed in nearshore (>20 m depth) and channel habitats (>60 m depth) throughout much of the spring and summer (Fig. 3). Phytoplankton blooms can lead to increased pH^13,41^ particularly in stratified systems like Puget Sound and Hood Canal^42^ in which the effect of primary productivity may be magnified by spatially decoupled productivity and decomposition^23,43^. Nearshore and channel habitats that experience seasonal stratification had elevated and less variable pH during spring and summer compared to well mixed areas like the coastal estuaries and sites within the Central Salish Sea (Fig. 1). The correlation of pH to chlorophyll and %DO concentration provides further evidence that distributed microalgae are ecosystem engineers of carbonate chemistry at large scales, a suggestion corroborated by observations of a transition from aragonite undersaturation to supersaturation concomitant to seasonally elevated primary productivity from numerous locations, including the West Antarctic Peninsula^44^, North Atlantic^13^, western Arctic Ocean^23^, Dutch Coastal zone^28^, the St. Lawrence Estuary^45^ and the Busan coast in South Korea^46^.

Quantifying connections to broader oceanographic processes is important to predict patterns of local pH variation^47^. Seasonal upwelling, freshwater inputs from rivers, and temperature changes putatively drive variation of carbonate chemistry in many coastal systems^48,49^. The influence of upwelling in Washington’s coastal estuaries dissipates quickly with distance into the bay as biological processes begin to modulate variability^30^. Patterns of pH variation consistent with the influence of upwelling were not observed on a large scale in this study; instead, pH patterns were more parsimoniously related to diel, seasonal and habitat-driven changes in ecosystem metabolism. Freshwater reduces seawater buffering capacity, which should result in lower pH^1^. Weak negative effects of decreased salinity on pH were observed, and may be localized in coastal ecosystems of NE Pacific Ocean, for example, contributing to the low mean pH observed in the Coastal Estuaries in this study. The observations of salinity <15 that were excluded from this analysis represented ~6% of the dataset, primarily in the Coastal Estuaries; the overall small relative effect of salinity is representative of the biologically-driven pH variation in this system. Negative effect of decreased salinity may also be counteracted by indirect effects of freshwater input on density stratification that result in increased primary productivity^24^. Contrary to predictions based on physical changes alone, temperature was positively correlated to pH, even when controlling for seasonal and subregional differences. However, this positive correlation was not evident at temperatures greater than ~18 °C. The transition to heterotrophy at peak summer temperatures has been observed to drive pH and %DO decreases in many estuaries^10,25^ and highlights the importance of considering the interaction of abiotic and biotic factors across scales. The small physical effects compared to the effect of ecosystem metabolism suggests that oceanographic control of carbonate chemistry in this ecosystem is indirect, and largely a function of physical effects on primary productivity and respiration.

Studies in the open ocean have observed spatial variability in long-term trends of pH, but the trends generally correspond to increased atmospheric CO_2_^34,50^. We documented variable rates of pH change among sites within a single ecosystem that ranged up to 10 times greater than the atmospheric CO_2_ trend. Furthermore, many sites had non-significant negative trends and one site in Willapa Bay within the Coastal Estuaries had a positive, but non-significant, trend of pH (Fig. 2a). High variability in the rate of pH change among sites within the same ecosystem, and even within the same subregion, provides strong evidence for local ecosystem drivers of pH fluctuations^8,10,27,28^. High spatial variability, particularly over tideflats, suggests models based on mid-channel monitoring may inaccurately predict conditions on the benthos due to different ecological contexts. Across sites, decadal-scale decreases of %DO and increasing heterotrophy are further support a biological driver of declining pH. The long-term pH and %DO trends generally fell along the expected relationship between pH and %DO, but some sites deviated from this pattern (Fig. 2c). Deviations may be driven by methodological factors such as years of sampling or physical variables such as the diffusion rates of pH and %DO at extremes of the observed range. Oxygen and CO_2_ solubility differ by an order of magnitude, a factor that may be important in surface waters with large deviations from atmospheric equilibrium of pH (e.g. ±1.3 pH) and DO (30–200% saturation) driven by biological processes^51^. These extremes lead to dramatic gradients in air-sea gas concentrations that could explain poor correlation between pH and DO at sites with high variability of these parameters^52^.

Ecological factors like the spatial coupling or de-coupling of biomass production and decomposition can furthermore play a large role in the observed relationship between pH and dissolved oxygen, particularly in high productivity, stratified nearshore regions. The theoretical relationship between primary production and respiration may not accurately predict empirical observations, particularly at longer timescales when variable rates of gas exchange and mixing water masses alter effects of local biological processes^10^. The movement and mixing of water masses can contribute to decoupling of biomass production and decomposition, leading to poor correlation between local pH and %DO. Much of this region experiences large tidal exchanges (>3 meters), seasonal wind-driven mixing and estuarine circulation such that measurements taken at monthly intervals may encounter water masses with different histories. Long-term monitoring programs are critical for establishing baselines and identifying anomalies of exposure to pH and dissolved oxygen. This study provides evidence that the seasonal changes to local ecosystem metabolism dominate other sources of variability in this system, but more research at different scales is required to fully understand the influence of physical and biological factors on pH dynamics across ecosystems.

The atmospheric equilibrium model of ocean acidification was inadequate for predicting recent (25-year) changes in this productive nearshore ecosystem: surface water was net heterotrophic over the study period and rarely in equilibrium with the atmosphere. Predicting exposure of marine organisms to stressful carbonate chemistry cannot exclusively follow global-scale biogeochemistry trends and will be improved through integration of organisms as drivers, not just responders, of change. Acknowledging the biological influence on pH variability allowed us to link ecologically-driven pH changes to temporal and spatial heterogeneity of CO_2_ exchange with the atmosphere in response to net organic matter production (Fig. 1, estimated CO_2_ sink = green) and decomposition (Fig. 1, CO_2_ source = purple)^53^. This framework provides the basis for integrating studies of physiological, community, and food web ecology with biogeochemical studies^54^. Many food webs in coastal habitats include reciprocal linkages from the water column to the benthos, particularly when phytoplankton serve as food to suspension-feeders but may compete for light with macrophytes. The quality and quantity of suspended particles derives in part from the balance of phytoplankton production and decomposition, and these suspended particles in turn influence turbidity, light penetration, and food quality for suspension feeders. High concentrations of detrital particulates in the water column simultaneously limit light penetration and subsequent photosynthetic uptake of CO_2_ by macrophytes^11^, while contributing CO_2_ from microbial degradation of detrital particulates^53^. The sensitivity of pH to net ecosystem metabolism suggests that the growing network of pH sensors around the world contributes to monitoring long-term pH changes, while providing an unprecedented ability to observe ecosystem function in near-real time and improve the resolution of carbon flux dynamics from a diversity of habitats, a biogeochemical research priority^55^. The diversity of nearshore habitats covered by our study adds to those such as riverine^1^, salt marsh^56^, shallow estuarine systems^53,57^ and tidepools^16^ in which ecosystem metabolism modulates local pH variability and response to global climate change.

## Materials and Methods

### Spatial and temporal variation of pH and %DO

Patterns of seawater pH (NBS scale) and the relationship to abiotic and biotic ecological factors were quantified in the coastal waters of Washington State at 83 sites spanning 25 years from 1991–2015 (Fig. 1). Sites were distributed throughout five subregions including the Coastal Estuaries (Grays Harbor and Willapa Bay), Hood Canal, South Puget Sound (south of the Tacoma Narrows), Puget Sound (main basin and Whidbey basin) and Central Salish Sea (sites north of Admiralty Inlet and in eastern Strait of Juan de Fuca). Environmental data were collected by the Washington state Department of Ecology (80 sites, WA-DOE) (fortress.wa.gov/ecy/eap/marinewq/mwdataset.asp) and the Padilla Bay National Estuarine Research Reserve (3 sites in Central Salish Sea, PB-NERR) (cdmo.baruch.sc.edu/get/export.cfm). Data used in this analysis met rigorous calibration and quality control standards. Data from WA-DOE include monthly water column profiles sampled at 0.5 m depth increments spanning the time period from October 1991 – October 2015^58^. Temperature (°C), salinity, dissolved oxygen (DO, mg L^−1^, % saturation) and pH (NBS scale) data were measured with SBE sensors (Sea-Bird Electronics, Inc.) throughout the study period. WA-DOE monitors 38 permanent ‘core’ sites and 45 rotating sites; all data were used for seasonal summaries and modeling analyses, whereas long-term trends were assessed with core sites owing to longer periods of observation. PB-NERR data were collected from moored, stationary sensors at 30 min (1995–2007) or 15 min (2007 – October 2015) intervals. Temperature (°C), salinity, dissolved oxygen (DO, mg L^−1^, % saturation) and pH (NBS scale) data were measured using an YSI 6600 multiprobe sonde (YSI, Inc.). These three sites were included in the ‘core’ sites for long-term analyses.

The methods of data collection differed in that a single time point was associated with multiple depths at WA-DOE sites, but only a single depth at PB-NERR sites. To make observations from these datasets comparable, one observation representing a single time and depth was randomly selected for each month from the 304,989 observations from the WA-DOE and PB-NERR monitoring sites resulting in 5994 observations. For PB-NERR sites, the monthly sample was selected during daytime hours between 0900 and 1700 local time. For WA-DOE sites, one measurement near the surface of each monthly depth profile (0.5 to 4.5 m) was selected at random. Observations were filtered to exclude measurements of salinity <15 to restrict error associated with predicting carbonate chemistry parameters below this level^59^. The subsampling method provided a data set with appropriate weighting (one sample per site and month each year) but used a small fraction of the data. To ensure the random sample used in the following analyses gave robust results and were not biased by the subsampling method, we compared long-term trends of pH and dissolved oxygen calculated with independent subsamples of the full ‘core’ site dataset (250 iterations).

#### Multi-decadal pH and %DO trends

Long-term temporal trends over the 25-year study period were assessed for both pH and %DO saturation with data from the 38 core sites (N = 4961 observations) using a linear mixed-effects model structure (LME) with year as explanatory factor and subregion and season as random effects. These smaller-scale factors were considered random effects here, where the focus was on long-term trends, but were explicitly considered for their contributions to pH variability in subsequent analyses (see below). Separate linear regressions were run for the upper-quartile and lower-quartile of the annual pH distribution. In addition to the region-wide analysis, trends at each core site were assessed separately using linear models. Not all sites were sampled in every month.

#### Seasonal and spatial variability of pH and %DO

Patterns of variability of mean pH and %DO were investigated using linear models with multiple spatial (site, habitat, subregion) and temporal scales as predictor variables. Habitats included tide flat (22 sites; <~17 m depth, sampled over tidal flat or predominantly well-mixed shallow inlets influenced by tidal flats), nearshore (38 sites; water depth was 17–60 m and close to shore or within a confined embayment) and channel (23 sites; in main channels with water depth >60 m). Homogeneity of variance of pH among habitats was tested in each season using Bartlett’s test. Seasons were defined as Winter = December, January, February, Spring = March, April, May, Summer = June, July, August, and Fall = September, October, and November.

In addition to this statistical modeling, the extent to which surface waters in the region were out of equilibrium with the atmosphere was visualized by developing a metric to demonstrate the metabolic effect on pH, analogous to %DO. Metabolic effect on pH was calculated by subtracting the predicted ‘atmospheric equilibrium’ pH based on physical conditions from the observed pH. ‘Atmospheric equilibrium’ pH was calculated using local temperature, salinity, estimated alkalinity from salinity *sensu* (^60^) and *p*CO_2_ in the ‘seacarb’ package in R^59^. We assumed that *p*CO_2_ concentration was in equilibrium with the mean annual atmospheric concentration obtained from NOAA (www.esrl.noaa.gov/gmd/ccgg/trends/). The marine source water that feeds Puget Sound and Hood Canal is characterized by mean *p*CO_2_ concentration near 700 µatm^61^. Source water for the Coastal Estuaries may include upwelled deep North Pacific water^30^. Seasonal variation of the source water to these subregions is difficult to constrain because of limited observations. We therefore used atmospheric CO_2_ concentration as the equilibrium point for the calculation of the metabolic effect because (a) most ocean acidification model projections consider surface oceans to be in equilibrium with the atmosphere, (b) surface waters of this region are in contact with the atmosphere for timescales required to reach equilibration with the atmosphere^62^, (c) this assumption simplifies the treatment of different water bodies that vary in their marine and freshwater sources, and (d) comparing *p*CO_2_ to atmospheric CO_2_ allows for a simple indicator of source-sink dynamics. Using the mean Strait of Juan de Fuca water (~7.8) as the equilibrium point would not effect the analyses in this paper, it would simply amplify pH anomalies by ~+0.2 units without changing seasonal patterns. The predicted pH was subtracted from the observed pH so that positive deviations (CO_2_ sink) indicate the influence of primary productivity while negative deviations (CO_2_ source) indicate the influence of respiration either locally or transported from outside the system.

To investigate potential consequences of pH variation for calcifying organisms, aragonite saturation state was calculated in ‘seacarb’ with observed pH, temperature, salinity and alkalinity predicted from salinity. The proportion of observations above saturation (Ω > 1) was calculated for each month. The probability of saturation metric is a conservative estimate of stressful carbonate chemistry conditions as organisms have been observed to be sensitive to aragonite saturation states of <1.6^36^, and states <1 mark the physical threshold of aragonite dissolution.

#### Time of day

Small-scale (within-day) temporal variability of pH and %DO associated with time of sampling was quantified using linear mixed effects models with random effects of site and season within year (N = 5994).

### Drivers of pH variability

Analyses and visualizations described in prior sections tested for spatial and temporal variation of pH and %DO at different scales. Here, %DO is incorporated as a potential predictor of pH, along with abiotic and climatic factors considered likely drivers. Subregion and season were identified as random effects following methods in^63^ to constrain spatial and temporal variation. Explanatory variables that were correlated to each other (r > 0.6) were excluded from the same analysis. Temperature, salinity and %DO data sampled simultaneous to pH, and monthly atmospheric CO_2_ concentration were explanatory factors, excluding interactions. Response and explanatory variables were normalized (mean = 0, SD = 1) to allow direct comparison of variables with disparate ranges of variation. Chlorophyll (Chl) was not sampled at PB-NERR and not available for many WA-DOE sites and therefore excluded from the full analysis. A supplementary analysis was conducted using the subset of 3123 observations with Chl. All mixed effects models were conducted with ‘lmer’ in R^64^, and significance of fixed effects determined with likelihood ratio tests.

## Supplementary information

Supplementary information
