# Supplementary material for: Ecosystem metabolism drives pH variability and modulates long-term ocean acidification in the Northeast Pacific coastal ocean
Source: Sci Rep. 2019 Jan 30;9:963. doi: 10.1038/s41598-018-37764-4 (PMC6353961; doi:10.1038/s41598-018-37764-4)
Supplement: Supplementary file 1 — Supplementary information [file 41598_2018_37764_MOESM1_ESM.pdf]

Ecosystem metabolism drives pH variability and modulates long-term ocean acidification in the Northeast Pacific coastal ocean

Alexander T. Lowe[1,2*], Julia Bos[3], and Jennifer Ruesink[2]

[1]Current address: Tennenbaum Marine Observatories Network, Smithsonian Institution, 647 Contees Wharf Road, Edgewater, MD 21307

[2]Department of Biology, University of Washington, 24 Kincaid Hall, Seattle, WA, USA 98195

[3]Washington Department of Ecology, 300 Desmond Dr. SE, Lacey, WA, USA 98503

*Corresponding author phone: 1-206-697-2765, email: lowea@si.edu

Supplementary Table 1. Locations and descriptions of sampling sites.

Site	Subregion	Habitat	Sampling	Max Depth (m)	Latitude	Longitude
GYS004	Coastal Estuary	Tideflat	Core	24	46.9779	-123.7846
GYS008	Coastal Estuary	Tideflat	Core	7	46.9373	-123.9132
GYS009	Coastal Estuary	Tideflat	Rotating	16.5	46.9645	-123.9496
GYS015	Coastal Estuary	Tideflat	Rotating	16.5	46.9229	-124.0768
GYS016	Coastal Estuary	Tideflat	Core	13	46.9534	-124.093
WPA001	Coastal Estuary	Tideflat	Core	12	46.6873	-123.7499
WPA003	Coastal Estuary	Tideflat	Core	10.5	46.704	-123.8374
WPA004	Coastal Estuary	Tideflat	Core	18.5	46.6868	-123.9735
WPA006	Coastal Estuary	Nearshore	Core	21.5	46.5454	-123.9802
WPA007	Coastal Estuary	Tideflat	Core	18	46.4532	-124.0096
WPA008	Coastal Estuary	Tideflat	Core	19.5	46.4632	-123.9413
WPA113	Coastal Estuary	Tideflat	Core	12	46.644	-123.993
HCB003	Hood Canal	Channel	Core	162	47.5379	-123.0096
HCB004	Hood Canal	Nearshore	Core	55.5	47.3562	-123.0249
HCB007	Hood Canal	Nearshore	Core	37	47.3981	-122.9296
HCB008	Hood Canal	Channel	Rotating	112	47.7533	-122.745
HCB009	Hood Canal	Channel	Rotating	97	47.6883	-122.75
HCB010	Hood Canal	Channel	Core	103	47.67	-122.82
PGA001	Hood Canal	Nearshore	Rotating	22.5	47.8398	-122.5813
ADM001	Central Salish Sea	Channel	Core	153	48.0298	-122.6179
ADM002	Central Salish Sea	Channel	Core	97	48.1873	-122.843
Bayview	Central Salish Sea	Tideflat	Core	4.5	48.4961	-122.5021
BLL009	Central Salish Sea	Nearshore	Core	31	48.6859	-122.5996
BLL011	Central Salish Sea	Nearshore	Rotating	24	48.7332	-122.5846
DIS001	Central Salish Sea	Nearshore	Rotating	42.5	48.0181	-122.8479
DRA002	Central Salish Sea	Nearshore	Rotating	23	48.9832	-122.763
DUN001	Central Salish Sea	Nearshore	Rotating	19.5	48.1731	-123.1146
EAS001	Central Salish Sea	Nearshore	Rotating	34	48.6429	-122.8835
FID001	Central Salish Sea	Tideflat	Rotating	16.5	48.5126	-122.5952
FRI001	Central Salish Sea	Nearshore	Rotating	19.5	48.5382	-123.013
FSH001	Central Salish Sea	Tideflat	Rotating	5.5	48.5098	-122.918
Gong	Central Salish Sea	Nearshore	Core	20	48.5583	-122.5725
GRG002	Central Salish Sea	Channel	Core	205.5	48.8082	-122.9541
JDF005	Central Salish Sea	Nearshore	Rotating	39.5	48.0609	-123.031
JDF007	Central Salish Sea	Nearshore	Rotating	29	48.0484	-123.0096
LOP001	Central Salish Sea	Nearshore	Rotating	16	48.5132	-122.8513
PAH003	Central Salish Sea	Nearshore	Rotating	22.5	48.1348	-123.4607
PAH008	Central Salish Sea	Nearshore	Rotating	19	48.1215	-123.3513
Ploeg	Central Salish Sea	Tideflat	Core	4.5	48.5563	-122.5308
PTH005	Central Salish Sea	Nearshore	Core	36.5	48.0831	-122.7646

RSR837	Central Salish Sea	Channel	Rotating	60	48.6165	-122.763
SEQ002	Central Salish Sea	Nearshore	Rotating	31.5	48.0765	-123.018
ADM003	Puget Sound	Channel	Core	214	47.879	-122.4832
CMB003	Puget Sound	Channel	Core	158.5	47.2904	-122.4501
CMB006	Puget Sound	Nearshore	Rotating	53.5	47.2615	-122.4373
DYE004	Puget Sound	Nearshore	Rotating	28.5	47.6223	-122.6896
EAG001	Puget Sound	Nearshore	Rotating	20.5	47.6217	-122.5017
EAP001	Puget Sound	Channel	Core	212.5	47.417	-122.3804
ELB015	Puget Sound	Channel	Core	131.5	47.5965	-122.3696
HLM001	Puget Sound	Nearshore	Rotating	53.5	48.0637	-122.5332
PMA001	Puget Sound	Nearshore	Rotating	51.5	47.7348	-122.5346
PNN001	Puget Sound	Nearshore	Rotating	31	48.2309	-122.6757
POD006	Puget Sound	Tideflat	Rotating	16	47.7148	-122.6346
POD007	Puget Sound	Tideflat	Rotating	6	47.7332	-122.6513
PSB003	Puget Sound	Channel	Core	110	47.6598	-122.4429
PSS008	Puget Sound	Nearshore	Rotating	37.5	47.9815	-122.2235
PSS010	Puget Sound	Channel	Rotating	109.5	47.965	-122.2633
PSS019	Puget Sound	Channel	Core	107	48.0109	-122.3013
QMH001	Puget Sound	Nearshore	Rotating	21.5	47.3798	-122.4662
QMH002	Puget Sound	Tideflat	Rotating	13	47.3965	-122.4429
SAR003	Puget Sound	Channel	Core	150	48.1076	-122.4915
SIN001	Puget Sound	Nearshore	Core	18	47.5493	-122.6435
SKG001	Puget Sound	Nearshore	Rotating	29.5	48.3957	-122.4896
SKG003	Puget Sound	Nearshore	Core	25	48.2965	-122.4896
SUZ001	Puget Sound	Channel	Rotating	107	48.1351	-122.3707
BML001	S Puget Sound	Tideflat	Rotating	14	47.3776	-122.6337
BUD002	S Puget Sound	Tideflat	Rotating	14	47.0515	-122.9063
BUD005	S Puget Sound	Nearshore	Core	20	47.092	-122.9182
CRR001	S Puget Sound	Channel	Core	110	47.2765	-122.7096
CSE001	S Puget Sound	Channel	Core	66.5	47.2645	-122.8443
CSE002	S Puget Sound	Nearshore	Rotating	24.5	47.3532	-122.8146
DNA001	S Puget Sound	Channel	Core	51.5	47.1615	-122.8718
ELD001	S Puget Sound	Nearshore	Rotating	23	47.1062	-122.9499
ELD002	S Puget Sound	Tideflat	Rotating	15	47.0962	-122.9754
GOR001	S Puget Sound	Channel	Core	171	47.1832	-122.6346
HND001	S Puget Sound	Nearshore	Rotating	25.5	47.1512	-122.8343
NSQ001	S Puget Sound	Nearshore	Rotating	33	47.1123	-122.6985
NSQ002	S Puget Sound	Channel	Core	111	47.1673	-122.7882
OAK004	S Puget Sound	Nearshore	Core	28	47.2134	-123.0777
PCK001	S Puget Sound	Nearshore	Rotating	22	47.2484	-122.9249
STL001	S Puget Sound	Channel	Rotating	122.5	47.1848	-122.6112
TOT001	S Puget Sound	Nearshore	Rotating	31.5	47.1643	-122.9646
TOT002	S Puget Sound	Tideflat	Rotating	17	47.1215	-123.0213

Supplementary table 2. Results of linear mixed effects model comparing pH variation to metabolic (%DO), biological (chlorophyll), physical (temperature and salinity) and climatic (atmospheric CO_2) drivers. (see methods for details, briefly: LME model with 3123 observations scaled to (x-μ)/SD. Fixed effects shown, random effects = Subregion + Seas.

Variable	Estimate	SE	X^2	*p*
DO % Saturation	0.598	0.016	1141.00	<0.001
Atmosphere CO_2	-0.163	0.012	165.76	<0.001
Temperature	0.127	0.020	40.58	<0.001
Salinity	0.047	0.014	11.04	<0.001
Chlorophyll	0.042	0.014	8.81	0.003
